# Formation of the 2015 Shenzhen landslide as observed by SAR shape-from-shading

**DOI:** 10.1038/srep43351

**Published:** 2017-03-03

**Authors:** Chisheng Wang, Qingquan Li, Jiasong Zhu, Wei Gao, Xinjian Shan, Jun Song, Xiaoli Ding

**Affiliations:** 1 Shenzhen Key Laboratory of Spatial Smart Sensing and Services and the Key Laboratory for Geo-Environment Monitoring of Coastal Zone of the National Administration of Surveying, Mapping and GeoInformation, Shenzhen University, Shenzhen, Guangdong, China; 2 Shenzhen Integrated Geotechnical Investigation and Surveying Co., Ltd., Shenzhen, China; 3 State Key Laboratory of Earthquake Dynamics, Institute of Geology, China Earthquake Administration, Beijing, China; 4 The Department of Land Surveying and Geo-Informatics, The Hong Kong Polytechnic University, Hung Hom, Kowloon, Hong Kong, China

## Abstract

The time-series topography change of a landfill site before its failure has rarely been surveyed in detail. However, this information is important for both landfill management and early warning of landslides. Here, we take the 2015 Shenzhen landslide as an example, and we use the radar shape-from-shading (SFS) technique to retrieve time-series digital elevation models of the landfill. The results suggest that the total filling volume reached 4,074,300 m^3^ in the one and a half years before the landslide, while 2,817,400 m^3^ slid down in the accident. Meanwhile, the landfill rate in most areas exceeded 2 m/month, which is the empirical upper threshold in landfill engineering. Using topography captured on December 12, 2015, the slope safety analysis gives a factor of safety of 0.932, suggesting that this slope was already hazardous before the landslide. We conclude that the synthetic aperture radar (SAR) SFS technique has the potential to contribute to landfill failure monitoring.

On December 20, 2015 (UTC 03:40, local time 11:40), a landslide occurred at the Hongao construction waste dump, Shenzhen (Fig. 1), which is a major financial center in southern China that has experienced rapid growth in recent decades. The landslide destroyed 33 buildings, killed 73 people, and left four missing^1^. This landslide was a typical waste landfill failure, with an impact area of 0.38 km^2^ and a length of 1100 m from south to north. The landfill site is located in the northern front of the central Dayan mountains. The natural slope of the mountains in this area is in the range of 25–35°.

There have been many landfill failure cases which were caused by the dumping of municipal solid waste, resulting in great economic loss and human casualties^3^,^4^,^5^. Human activities are often one of the main factors contributing to landfill failure. Rapid urbanization in China’s cities always comes with extremely high waste generation, and 79.3% of this waste goes to landfill^6^. Many landfill sites are now confronted by the problems of overloading caused by rapid waste generation, which brings a high risk of future landslides. Quantifying the potential risks can help to minimize the threat. An extensive review and comparative case studies of the methods for monitoring and mitigating the risk of landslide can be found in Malehmir *et al*.^7^. Since waste disposal takes place at landfills every day, regular analysis of the landfill stability is necessary to prevent landslide disasters. Landfill topography is the basic data used in slope stability analysis. However, field surveys are labor-intensive and provide only a sparse coverage. For example, there are more than 150 landfill sites in Shenzhen^8^, and periodically surveying the topography of each site is not realistic.

Synthetic aperture radar (SAR) data could be the best alternative data source to solve the problem. SAR data are an important data source for earth observation, having no limits to time and atmospheric conditions. SAR shape-from-shading (SFS) is a technique that can be used to estimate surface topography from a single SAR image and some ground control points (GCPs). It is based on the principle that radar backscatter is related to satellite incidence geometry^9^. With the SFS technique, the relationship is first used to solve the surface slopes, and then the surface topography can be constructed from these slopes and the GCPs^10^. This technique was first proposed decades ago as “radarclinometry”^11^. Its main advantage is that it requires only a single image. However, the accuracy of SFS is limited since radar scatter also depends on other non-topographic surface properties, such as roughness and composition^12^. The accuracy of the SFS technique is not guaranteed if these issues are not properly considered. Therefore, to date, the SFS technique has been routinely applied only in certain fields^13^,^14^.

The material of a waste landfill surface is normally composed of the clay residue produced from city construction, which gives a uniform appearance to the landfill. Therefore, SAR-SFS can be expected to produce a satisfactory accuracy in topography extraction. Witnessing the height growth of a waste dump is rare because field surveying is not often carried out before a landslide. Even if some surveying work had been done preceding the event, the spatial coverage is likely to be limited. However, the SAR-SFS technique could provide the opportunity to capture the progress of the landfill and to help prevent landslide disaster.

In this work, we perform a SAR-SFS time-series analysis of 21 Cosmo-SkyMed images of the 2015 Shenzhen landslide. The accuracy of the SAR-SFS digital elevation model (DEM) is examined by comparing it with an external contour map and field survey results. Based on the derived time-series SAR-SFS DEMs, we further investigate the main cause of this disaster and explore the possibility of regular landfill stability analysis based on the SAR-SFS technique.

## Activity in the Hongao landfill

**Figure 2:**
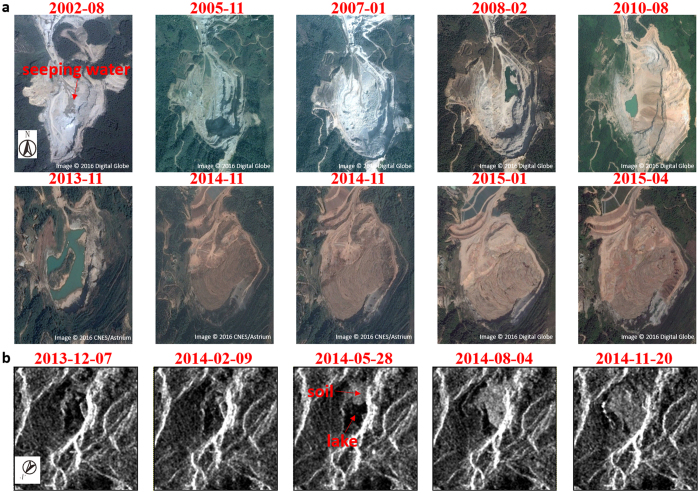
Evolution of the Hongao landfill site. (**a**) High-resolution optical satellite images (image data: Google, DigitalGlobe, CNES/Astrium). (**b**) Cosmo-SkyMed SAR images (purchased under license from the Italian Space Agency). In order to better visualize the topography, the SAR images are plotted in SAR coordinates rather than geographic coordinates.

The Hongao landfill site was formerly a quarry where stone was excavated over a number of years. A series of optical satellite images from Google Earth (Fig. 2a) reveals the transformation of the quarry and the formation of an open pit. The images show that the quarry was still active from 2002 to 2010, during which time the excavation area was moved from west to east, and the land cover was mainly composed of bare soil generated by stone excavation. Meanwhile, some traces of water seeping from the rocks can be seen in the image captured in August 2002 (Fig. 2a). As shown in the next series of images, the seeping water contributed to forming small lakes in the quarry, along with the rainfall.

In 2013, the quarry was abandoned and vegetation started to reclaim the bare land. The lakes in the quarry subsequently increased in size. The government then approved the establishment of a landfill site in February 2014. Comparing the images acquired in November 2013 and November 2014, the changes are significant. The high temporal resolution of the Cosmo-SkyMed radar images gives more details about the landfill process during this period. The landfill started in February 2014, and can be clearly seen in the following images. Soil and water both existed during the landfill construction period, suggesting that no effective drainage was undertaken in the quarry before waste dumping started (Fig. 2b). By August 2014, the lakes had disappeared as a result of the waste filling.

### SAR-SFS measurements and error analysis

The SAR-SFS technique can generate a DEM using only a single SAR image. After collecting 27 Cosmo-SkyMed radar images from December 2013 to January 2016, with a time resolution of about one month (Supplementary Table 1 and Supplementary Fig. 1), we undertook a time-series analysis of the dynamic DEM change over the landfill period. Since SFS greatly depends on the assumption of uniform surface properties^12^,^15^, six images obtained before June 2014 with large areas of water were eliminated to avoid any measurement error (Supplementary Fig. 2). As SAR-SFS is based on the integration of estimated slopes, an external DEM was used to calibrate the measurements.

**Figure 3:**
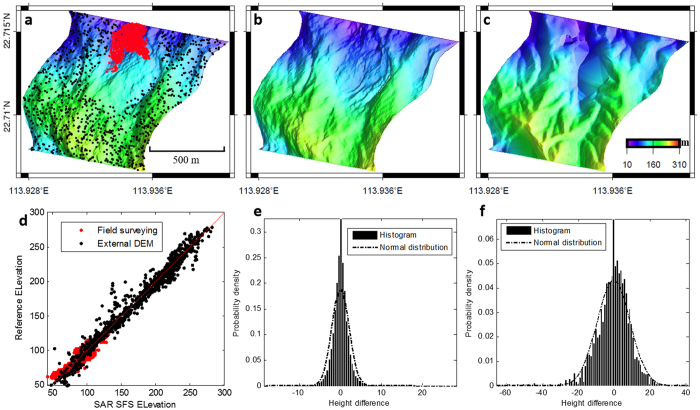
SAR-SFS result validation. (**a**) SAR-SFS topography result for December 13, 2015. The 1000 randomly selected black points are used to compare with an external DEM generated before the landslide. The red points are used to compare with the field survey results after the landslide. (**b**) SAR-SFS topography result for January 14, 2016. (**c**) The DEM interpolated from a 1:1000 contour map. (**d**) Comparison between the SAR-SFS results and the external datasets. (**e**) Histogram of errors from the intra-comparison of the 21 SAR-SFS DEMs for the 1000 randomly selected points. (**f**) Histogram of errors comparing the SAR-SFS results with the external datasets.

To validate the accuracy of the derived DEMs, the SAR-SFS DEMs were compared with each other, as well as with external datasets, which included a 1:1000 contour map obtained before the landslide, and some field investigation results from January 1, 2016, obtained after the landslide. Firstly, 1000 points were randomly selected outside the quarry, from the points not used for the calibration (Fig. 3a). The 1000 selected points were mostly distributed on the natural mountains around the quarry. The elevations of these points were deemed to be consistent over the observation period. The intra-comparison of the 21 SAR-SFS DEMs was implemented by calculating the standard deviation (STD) of the height changes (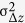) of the 1000 points, which takes the form of,


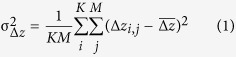


where 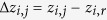 is the height change between the *j*th SAR-SFS DEM (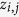) and the reference SAR-SFS DEM (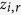) on the *i*th pixel, 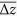 is the mean value of 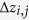, *K* = 1000 is the number of selected points, and *M* = 20 is the number of SAR-SFS DEMs used for the comparison (the first SAR-SFS DEM on June 17, 2014, was taken as the reference DEM).

The distribution of the SAR-SFS DEM height changes (Δ*z*) on the selected points is shown in Fig. 3e. This exhibits a Gaussian distribution centered on Δ*z* = 0 with an STD (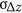) of 2.3 m (Table 1), suggesting a satisfactory internal consistency for the SAR-SFS results. Furthermore, the average heights of these points in the 21 SAR-SFS DEMs were compared with the 1:1000 contour map. For a better comparison, the contour map was converted to a triangulated irregular network (TIN) and then interpolated into a DEM (Fig. 3c). Meanwhile, the SAR-SFS DEM obtained on January 15, 2016 (Fig. 3b), was compared with the field survey results obtained after the landslide on January 1, 2016. These comparisons confirmed that the SAR-SFS DEM generally agrees well with the external datasets, with an STD (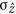) of 9.4 m (Fig. 3f, Table 1).

However, it should be noted that the STD (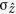) of 9.4 m is an estimated value. This value is affected by the uncertainties in both the SAR-SFS results and the external datasets. Assuming that the SAR-SFS DEM is independent of the external data, the estimated STD can be decomposed as,


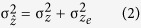


where 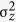 and 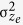 are the real uncertainties of SAR-SFS height (*z*) and external height (*z*_*e*_), respectively.

Since the external DEM was interpolated from a contour map, some interpolation errors may have existed. Meanwhile, because the field survey was undertaken 14 days after the SAR image was captured, the clean-up work during this period may have resulted in height discrepancies. Therefore, the real uncertainty of the SAR-SFS DEM (σ_*z*_) may in fact be much less than the calculated result (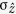
=9.4 m).

From the SAR-SFS heights, the corresponding volume change (V) of the Hongao landfill over time can be calculated. Based on the error propagation rules, we can also estimate the uncertainty of the calculated volume change (σ_*V*_). Following a previous study^16^, the formulas for the volume calculation and error estimation are given as,


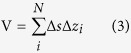



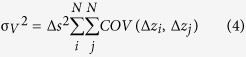


where Δ*s* is the area size for one pixel, Δ*z*_*i*_ is the height change of the *i*th pixel, *N* is the number of pixels used to calculate the volume change, and 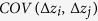 is the covariance between the height changes of the *i*th pixel and the *J*th pixel. The variance of the volume change strongly depends on the correlation of the height change measurements. When the height changes on pixels are completely independent, σ_*V*_ has a minimum value, which is given as 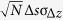, where 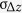 is the variance of the height change measurements. When the height changes are completely correlated, σ_*V*_ has a maximum value taking the form of 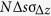.

**Figure 4:**
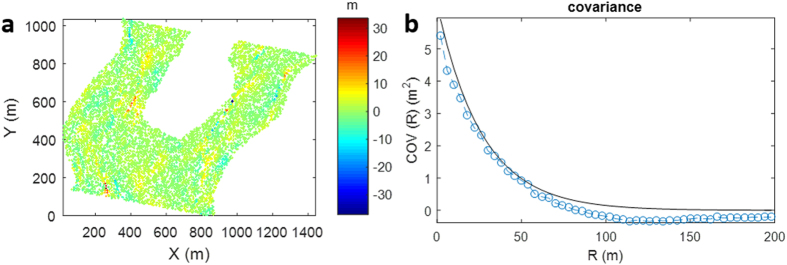
Error analysis of the volume change. (**a**) Difference between two SAR-SFS results on 10000 randomly selected points outside the landfill. (**b**) The calculated covariance values (blue circles) and fitted exponential covariance model (black line).

We randomly selected a DEM pair from the SAR-SFS results to calculate the covariance matrix. Assuming that there was no DEM change outside the quarry, the DEM difference on the surrounding points can be considered as the measurement error for the uncertainty estimation. Here, we randomly selected 10000 surrounding points to calculate the statistical information (Fig. 4a). The average covariance as a function of the distance is given in Fig. 4b. An exponential covariance model (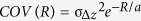, where *a* is the correlation length to be estimated) was used to fit the calculation. This covariance model can be used to compute the uncertainty of the volume change in the landfill area. In this case, the landfill contains 2059483 pixels, and each pixel covers an area of 0.3 m × 0.3 m. According to the spatial distribution of the pixels and the estimated covariance-distance function, the variance of the volume change was calculated to be 66,959 m^3^ (Table 1). This value is between the lower bound (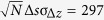 m^3^) and the upper bound (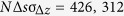 m^3^) for the volume change error.

### Topographic evolution of the Hongao Landfill

**Figure 5:**
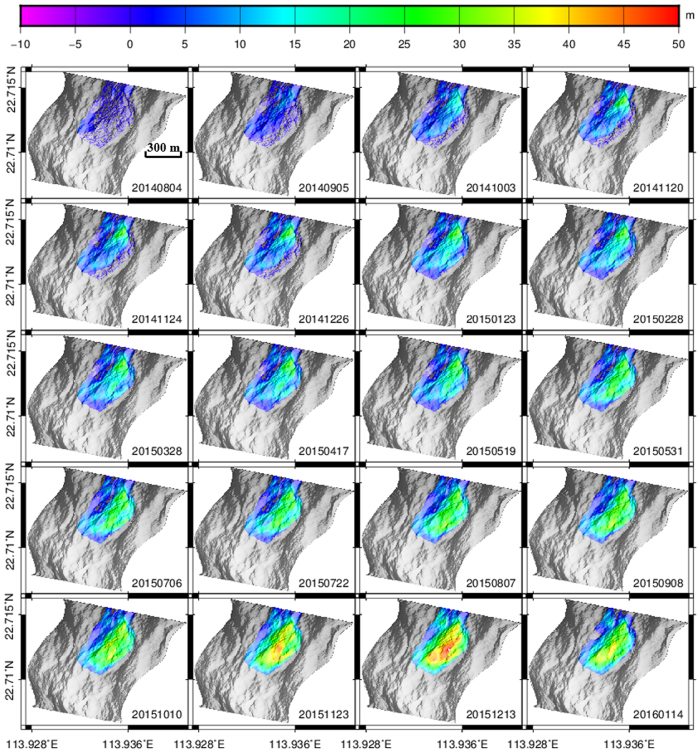
SAR-SFS time-series topography change referring to the DEM on June 17, 2014. The last map on January 14, 2016, after the landslide, refers to the DEM on December 13, 2015, and represents the soil volume sliding down in the landslide.

The time-series SAR-SFS results show that the volume of the landfill kept increasing from June 2014 (Fig. 5 and Supplementary Fig. 3). The size of the area that was increasing in volume was about 0.18 km^2^, which agrees with the size of the landfill area observed in the optical images. The greatest volume change was found at the east side of the landfill, where the largest elevation increase reached almost 50 ± 2.3 m. As observed in the high-resolution optical images, a road was constructed in the east to allow access for the slag car. The east part of the landfill is the area where the waste dumping started, and was therefore expected to collect the most construction waste, as revealed in the SAR-SFS results. From June 2014 to May 2015, waste filling mainly took place in the northeast since the northern area was low-lying and suitable for land filling. After May 2015, the increase in volume gradually transferred to the north. Finally, the waste dumped in the north was higher than the south, agreeing with the local terrain slope.

The accumulated volume in this quarry reached 4,074,300 ± 66,959 m^3^ by December 13, 2015. Five days after this, the landslide occurred. By comparing this figure with the SAR-SFS DEM on January 14, 2016, the volume of the landslide was calculated to be 2,817,400 ± 66,959 m^3^. The official accident investigation reports^1^ announced that 2,780,000 m^3^ of waste was shipped out after the landslide, which is close to the calculated landslide volume. The landslide area is in agreement with the landfill area, implying that only the landfill participated in the event.

**Figure 6:**
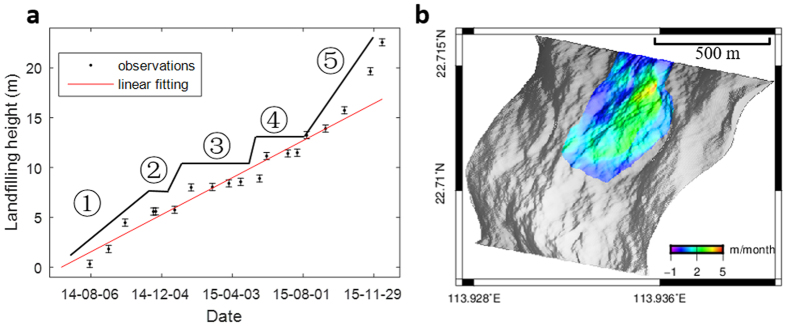
Mean landfill rate. (**a**) Mean landfill height as a function of time. The black lines and numbers indicate the five stages of landfilling. The error bar for average height increase is plotted according to its STD, which is calculated from 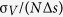. (**b**) Landfill rate distribution.

The average increase in height at the landfill points over time was plotted to explore the time variation of the landfill process. This shows that the waste dumping was rapid in the first 180 days from June 2014 to December 2014 (stage 1). After that, the waste dumping slowed down for the next 170 days from December 2014 to May 2015 (stages 2 and 3). As revealed by the investigation reports^17^, the landfill site was closed by the government on December 2, 2014, because of a missing environmental impact assessment. However, some slight volume changes can be witnessed during this period. When the environmental impact assessment report was submitted and approved, the waste dumping resumed. The landfill pace increased quickly again in the last 210 days from May 2015 to December 2015 (stages 4 and 5). After applying a linear fitting to the landfill change plot, the mean height change in the quarry was found to be 0.9 m/month (Fig. 6a). To investigate the spatial variation of the mean height change, this value was calculated for each landfill point. The results show that the elevation increase was mainly concentrated in the east, where the maximum mean height increase reached 3.5 m/month (Fig. 6b). Meanwhile, a number of points exceeded 2 m/month, which is an empirical upper threshold in landfilling to ensure engineering safety^18^.

### Slope safety analysis

**Figure 7:**
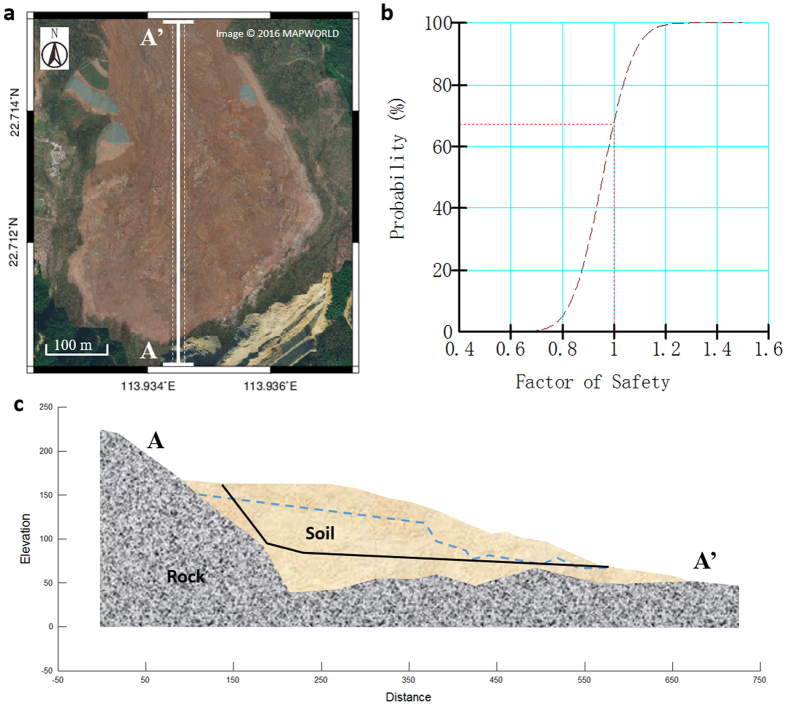
Slope safety analysis. (**a**) The location of the elevation profile is denoted by the white line. Points in the 10 m buffer region are averaged to form the longitudinal elevation profile. The background aerial image was obtained from www.tianditu.com. (**b**) The probability of failure defined as P(FOS < 1). (**c**) Landfill slope setting. The black line denotes the critical slip surface and the blue dashed line denotes the piezometric line. The figure was generated in Generic Mapping Tools (GMT 5.0 from http://gmt.soest.hawaii.edu/).

With the detailed topography obtained by the SAR-SFS technique, it was possible to build a numerical model based on the image data and undertake a slope safety analysis. A slope factor of safety (FOS) was calculated, which is defined as the ratio of the shear strength to the shear stress required to keep the slope stable. If the value of FOS is less than 1.0, the slope can be considered to be unstable. In the analysis, the surface elevation profile was set according to the SAR-SFS DEM for December 13, 2015 (Fig. 7). At this time, the landfill pit resembled a huge pond, which was surrounded by mountains to the east, west, and south, and extended mainly along the north–south direction. The elevation of the back edge of the landfill pit was 155 m and the front edge elevation was about 50 m.

According to the field investigation results^17^, the slopes were classified as soil and rock. Although these materials could be divided into more detailed groups, two major classes were selected since the cohesion and friction angles of the other materials were similar to the soil and rock classes. The upper layer was mainly described as a “sliding soil mass”, in which the complex composition consisted of local weathered soil (decomposed from sandstone, granite, and migmatite), construction waste (baked clay bricks and concrete bricks), as well as diluvial gravel and soil-rock mixtures abandoned by the quarry. The soil mass was described as soft and loose, with an average natural water content of around 22.6%. The degree of saturation varied from 60% to 95%, indicating uneven saturation and a wet state in this soil mass layer. Silty clay or sandy clay could be defined based on the relatively low plasticity index. Based on the results of consolidated quick shear tests^17^, the average values of the mechanical parameters of the soil mass were obtained as 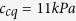 and 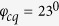 from a total of 43 samples. Referring to the direct shear test results^19^, the measured shear strength in these samples was 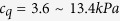 and 
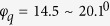
, and the average shear strength was 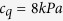 and *φ*_*q*_ = 18.8^0^. The residual shear strength of the disturbed soil samples with intercalated silt layers was determined as 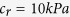 and *φ*_*r*_ = 17^0^. The exposed bedrock of the original pit was composed of moderately and slightly decomposed granite (i.e. MDG and SDG).

The hydrologic condition was an important factor in this landslide accident. As a result of the topographical properties of the quarry pit, the rainfall surface water of the surrounding slopes and the groundwater from inside the granite rock fractures gathered in the low elevation area. Furthermore, because of the relatively low permeability coefficient of the landfill soil (which is usually 2.05 × 10^−5^ to 1.89 × 10^−6^ cm/s)^17^ and the rapid soil loading rate, water accumulated within the landfill soil body throughout the entire landfill process and was maintained at a relatively high level. The piezometric water line defined in the analysis model followed the seep analysis results of a previous study^19^.

Finally, GeoStudio software was applied for the landslide stability analysis. The material properties, piezometric lines, and rock boundaries were set in the model according to the above descriptions, the SAR-SFS technique, and the results from the field investigation (Fig. 7 and Table 2). Because the large volume of upper landfill soil was filled and formed gradually from materials of relatively similar compositions, such as local weathered soil, construction waste, etc., it was necessary to simplify the upper landfill soil mass as one analytical object with uniform shear strength in the simulation model. In this situation, a translational failure mode was chosen as a reasonable calculation approach in the analysis. Furthermore, the parameters applied in the numerical simulation took the hydrologic condition and consolidation state into account. The slope safety analysis gave an FOS of 0.932, suggesting that this slope could have slid at any time, without external force.

Since uncertainties existed among the input material parameters, a probability test was applied to the slope safety analysis. The unit weight, cohesion, and friction angle of the upper soil were assumed to follow a Gaussian distribution and have STDs (σ) of 1 kN/m^3^, 1 kPa, and 1°, respectively. In total, 2000 Monte Carlo trials were run with unit weight, cohesion, and friction angle setting ranges of 5–15 kN/m^3^, 7–10 kPa, and 14.7–17°. The tests showed that the probability of failure (P(FOS < 1)) was 67.9% (Fig. 7b). Normally, the probability of failure should not be allowed to reach 2.3%, while a probability value of 16% can be defined as “hazardous”^20^. Therefore, this landfill slope was far more serious than “hazardous” before the landslide.

## Discussion

### Landfill monitoring with the SAR-SFS technique

The accuracy of the SAR-SFS results can be affected by several important factors, including the reflectance model, the inversion algorithm, and the height reconstruction. Firstly, in this study, for simplicity, we used a Lambertian reflectance model to describe the relationship between the image intensity and the surface slopes. As the waste in the Hongao landfill site was mainly composed of clay generated at construction sites, the surface composition could be considered as uniform. The Lambertian model could therefore provide a satisfactory approximation to the SAR image formation. However, there was still some inevitable discrepancy between the landfill SAR scattering and the Lambertian model, which may have resulted in some measurement errors. Secondly, in this study, we used the iterative minimization SFS algorithm to solve the surface slopes from the SAR images. This algorithm has been reported to be more robust and accurate than others^21^. However, no matter which algorithm is used, SFS is always an ill-posed problem, which means that many surface slope solutions can be derived from one SAR image. Although a smoothing constraint and integrability constraint are introduced to avoid abnormal solutions, the derived solution can still deviate from the true values. Finally, in this study, we converted slopes to heights in the frequency domain based on a Fourier transform. Calibration was required because the computational results were arbitrarily scaled and the average height value could not be recovered. For this study, a limitation of this step was that there were no landfill points that could be used for the calibration. Using the non-moving forest points outside the landfill, which had less uniform scattering characteristics, may have biased the results within the landfill.

The uncertainty of the SAR-SFS results in this study was estimated in three ways: intra-comparison, external comparison, and volume error calculation. The results suggested that the STDs of the errors for the height change, the height, and the volume change were 2.3 m, 9.4 m, and 66,959 m^3^, respectively. The discrepancy between the height change error and the height error can be explained on multiple grounds. First of all, the height change was obtained by differencing the height values of two SAR-SFS results. As some of the systemic errors of the SAR-SFS had been removed, the height difference error was relatively small. Secondly, the external datasets used for the validation were obtained at different times and may have contained inherent errors, so the estimated error may have deviated from the real value. As landfill monitoring is mainly concerned with height change, the estimated errors for the height change may be more appropriate for the uncertainty analysis. A similar accuracy of 2–4 m was reported in a previous SFS study^21^. Considering the rapid waste dumping in this landfill site, it is believed that the SAR-SFS results are able to capture the height changes.

Although the volume change is proportional to the height change, the volume change error and height change error do not exhibit a simple linear relationship, and this is greatly dependent on the spatial correlation of the SAR-SFS errors. The integration step of the SAR-SFS algorithm inevitably causes errors to propagate from one pixel to another pixel in the results. It is therefore not surprising to find a relatively large correlation length of 26.7 m in the SAR-SFS errors (Fig. 3b). The calculated STD turns out to be 66,959 m^3^, which is much larger than the lower bound of 297 m^3^ in the case of all the errors being independent. However, compared with the large landfill volume of 4,074,300 m^3^ in the one and a half years before the landslide, the volume error of SAR-SFS is still acceptable (less than 2%).

### Triggering mechanism

The 2015 Shenzhen landslide was unexpected and resulted in many unnecessary casualties. Understanding the mechanism of this landslide is important to avoid such a situation happening again. Normally, there are three major factors considered to contribute to landfill failures: slope gradient, waste compaction, and water pressure^22^,^23^.

High slope gradient can accelerate slope instability by increasing the shear force. The steeper the slope of the landfill, the more likely it is that a landslide will happen. However, the SAR-SFS result for December 13, 2015, suggested that the Shenzhen Hongao landfill had gentle slope gradients before its failure (Fig. 3a). The maximum slope was around 20°, which was much less than the approximately 45° slopes found in some similar landfill failures, such as the 1977 Sarajevo event, the 1993 Istanbul event, the 2000 Payatas event, and the 2005 Bandung event^24^. This implies that the slope gradient may not have been the main factor in the triggering mechanism of this event.

Waste compaction can significantly increase shear strength in landfills, and therefore reduce the likelihood of failure. The time-series SAR-SFS DEM results revealed that the landfill volume in the last stage (from April 2015 to December 2015) increased rapidly over time (Figs 5 and 6). Waste compaction is usually accompanied by a slow descending of the topography. However, this is inconsistent with the rapid volume increase of the landfill observed in the SAR-SFS results, implying that there was no waste compaction undertaken during this period. In many landfill failures, the lack of waste compaction has been considered to be an important contributory factor^25^.

High pore water pressure can reduce waste shear strength and cause instability and failure of a slope. The SFS DEMs show a steep and hilly topographical characteristic around the Hongao landfill site (Supplementary Fig. 3). This means that the surface water from the surrounding catchment area and water infiltration from the rock fractures would have flowed into the quarry pit. This phenomenon can be clearly observed in the optical images (Fig. 2). Without a well-designed drainage system, a high water head pressure would be formed over the years of landfill. Moreover, a rapid filling rate can also generate an excessive pore water pressure over a short period of time. Referring to some preloading project experience, the frequently used threshold for the soil filling rate in China is the maximum stress change rate, which is often designed as 1.2 kpa/d, and equal to 2 m/month when transferred to height change^18^. Stricter filling rates of 0.6–1.5 m/month have been given in projects in other countries^26^. According to the calculation of the time-series SAR-SFS DEM results, most of the landfill area exceeded the allowed rate, and the highest rate reached 3 m/month (Fig. 6b). Since the waste mainly consisted of a clay residue with high moisture and a low permeability coefficient, the rapid landfilling would have caused an increasing pore water pressure where the water in the clay could not be quickly extruded. Considering the topography, the rapid process of soil loading, and the composition of the landfill, it is not surprising that the field investigation found a high underground water level in the slope and a high water content in the soil^17^. Therefore, we believe that the high pore water pressure was the main reason for the landslide.

The slope safety analysis allowed us to further analyze the triggering mechanism and investigate the specific reason for this landslide. Based on the topography and the geotechnical parameters obtained from the SAR-SFS and the direct shear test results, the numerical model gave an FOS of 0.932, suggesting an unstable state for the Hongao landfill. By changing the model settings in the FOS calculation, it was possible to analyze their sensitivities to slope stability and infer the factors contributing the most to the instability of this landfill. We individually tested the sensitivity of topography, the piezometric line, cohesion, and friction angle, as shown in Supplementary Fig. 4. The results suggested that a change in slope gradient would not have greatly affected the FOS value. An elevation change of ± 5 m at the top of the slope would have resulted in a FOS change of around 0.01. This confirms the conclusion made above, i.e., the slope gradient was not the main factor in this event. In contrast, the FOS was found to be very sensitive to the piezometric line and friction angle. These two factors are strongly correlated to the water condition in the waste mass. In this landfill, the excess pore water pressure resulted in a high piezometric line and a low friction angle. The sensitivity testing showed that a decrease of ~5 m for the piezometric line or an increase of ~1.3° for the friction angle would have increased the FOS value to 1, making this landfill stable. This suggests that the most effective way to avoid similar catastrophic landfill failures would be to reduce the pore water pressure, which could be accomplished in many different ways, such as regularly compacting the waste, building a drainage system, or reducing the landfill rate.

## Conclusion

In summary, in this study, the time-series topography of the landslide at the Hongao landfill site was retrieved by the use of the SAR-SFS technique, before and after the event. We believe that this is the first time that a landfill failure has been witnessed with the SAR-SFS technique. A landfill safety analysis was then undertaken based on the SAR-SFS topography and the geotechnical parameters. The approach used in this study is a practicable way to obtain early warning of a landslide at a landfill site. Through analyzing the time-series SAR-SFS DEMs, we concluded that the absence of waste compaction and the high pore water pressure were the main reasons for the landslide at the Hongao landfill site.

## Methodology

### SAR-SFS algorithm

The iterative minimization SFS algorithm was used to retrieve the DEM from the SAR images. This method formulates the SFS problem as a cost minimization problem, and uses an iterative search approach based on the linearization of the reflectivity function^21^,^27^,^28^ to find the solution. Meanwhile, a smoothness constraint and integrability constraint are added in order to avoid physically or numerically abnormal solutions. The cost function (including the smoothness constraint) to be minimized is given as:


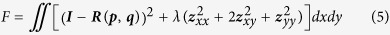


where ***I*** is the SAR brightness map (Supplementary Fig. 5a). 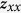, 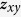, and 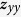 are the second partial derivatives of the estimated SAR-SFS DEM 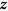. *λ* is the regularization parameter balancing the SAR intensity constraint and the smoothness constraint. 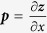 and 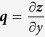 are the estimated surface gradients in the range and azimuth directions. ***R*** is the reflectivity function of the Lambertian surface, which takes the form of (Supplementary Fig. 5b):


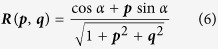


where *α* is the inclination angle of the SAR satellite.

Given the cost function described above, an iterative computation procedure was designed to recover the topography from the SAR magnitude image, following previous studies (Supplementary Fig. 6)^9^,^21^:

(1) Register all the SAR images and the reference DEM to a master SAR image.

(2) Calculate a reference reflectivity map **I**_*r*_ (simulated SAR) using Equation (6) with the reference DEM.

(3) Fit all the SAR magnitude images to the reference map by solving the equation:


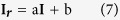


where **I** is the real SAR image magnitude, and a and b are the linear coefficients to be solved using the least-squares method. With the estimated coefficients 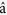 and 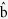, all the SAR magnitude images are normalized to the reference map.


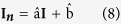


where **I**_***n***_ is the normalized SAR image.

(4) Initialize the zero surface gradients ***p*** and ***q***.

(5) Calculate the corresponding surface reflectance ***R***(***p***, ***q***) with the given ***p*** and ***q***.

(6) Perform partial differentiation of ***R***(***p***, ***q***) for each pixel:


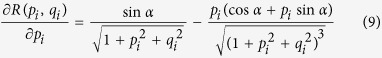



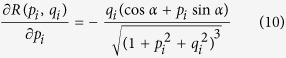


(7) Update the surface gradients from the (*k*)th to the (*k* + 1)th iteration using the partial derivatives and normalized SAR image **I**_***n***_:


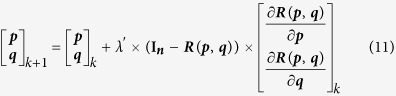


where *λ*′ is inversely proportional to regularization parameter *λ* in Equation (5) 9 (which was set as 0.3 in this study after many attempts).

(8) Apply median filtering of the new surface gradients, and return to step 3) if the convergence condition is not satisfied.

(9) Reconstruct the topography from the final surface gradients using a Fourier transform and the integrability constraint^29^:


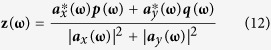



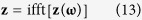


where 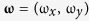 is the two-dimensional frequency coordinates, ***p***(**ω**) and ***q***(**ω**) are the Fourier transform of the final surface gradients, ***a***_*x*_(**ω**) and ***a***_*y*_(**ω**) are the Fourier coefficients of a discrete differentiation operator in x and y, and 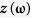 is the Fourier transform of the topography **z**.

(10) Calibrate the retrieved topography to an external DEM using polynomial fitting:


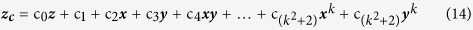


where 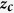 is the calibrated topography, ***x*** and ***y*** are the x- and y-coordinate vectors corresponding to the height vector 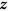, **c** is the polynomial coefficients estimated by fitting the retrieved topography **z** to the external DEM, and *k* is the order of polynomial fitting.

(11) Geocode the topography from SAR coordinates to geographical coordinates using the SAR geometry parameters and the corresponding calibrated elevation.

Please note that the mathematical operations on vectors (with boldface) in the above equations are all pixel-by-pixel operations. The convergence condition in step 8) could be a threshold on the iteration number or the cost function decreasing. In this study, we found that setting the maximum iteration number to 200 could obtain stable results for all the images. As the SAR intensity is normalized and the DC component (zero frequency) is invalid in Equation (12), the computational topography **z** based on the Fourier transform from step 9) is arbitrarily scaled and the average height value cannot be recovered^21^. Meanwhile, the low-frequency components of **z** may be misestimated because they are not well constrained by the image brightness. We therefore calibrated the results by a 2D polynomial model, which could compensate the height scale error, the constant height error, and the low-frequency components. Some GCPs from an external DEM were used to calculate the calibration coefficients. Because the points on the landfill varied greatly over time, we only used the surrounding points for the calibration. In this study, 1000 points distributed outside the landfill were randomly selected for the calibration. After many attempts, we found that *k* = 5 was an appropriate value for the polynomial model, which could give a satisfactory modeling of the low-frequency components.

### Factor of safety (FOS) calculation

We implemented the landfill stability analysis using GeoStudio software^30^. The Morgenstern-Price method^31^ was used to calculate the FOS. Since the water conditions played an important role in the 2015 Shenzhen landslide, the pore water pressure condition was also considered in the slope analysis and defined with a piezometric line. To avoid deriving physically inadmissible slip surfaces, a fully specified slip surface (following a previous study^19^) was selected to constrain the slip surface.

## Additional Information

**How to cite this article:** Wang, C. *et al*. Formation of the 2015 Shenzhen landslide as observed by SAR shape-from-shading. *Sci. Rep.* **7**, 43351; doi: 10.1038/srep43351 (2017).

**Publisher's note:** Springer Nature remains neutral with regard to jurisdictional claims in published maps and institutional affiliations.

## Supplementary Material

Supplementary Information

## Figures and Tables

**Figure 1:**
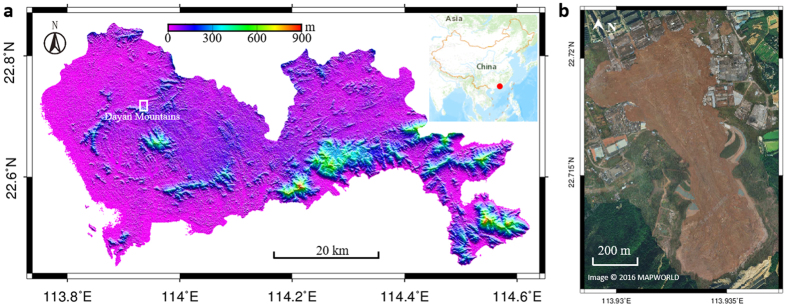
Geological and topographic setting. (**a**) Topography of Shenzhen (data from the ASTER GDEM^2^). The inset map shows the location of Shenzhen in China (data from ArcGIS online, purchased under license from Esri). The white box indicates the region mapped in b. (**b**) Aerial image of the landslide by unmanned aerial vehicle (data from www.tianditu.com). The figure was generated in Generic Mapping Tools (GMT 5.0 from http://gmt.soest.hawaii.edu/).

**Table 1:** Statistical information for the SAR-SFS DEM errors.

Intra-comparison (m)				External comparison (m)				Volume (m^3^)
max	min	mean	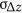	max	min	mean	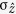	σ_*V*_
31.8	−22	0	2.3	37.7	−60.9	−0.43	9.4	66,959

**Table 2:** Geotechnical parameters of the input materials.

Material	Unit weight (kN/m^3^)		Cohesion (kPa)		Friction angle (deg)	
	range	fixed	range	fixed	range	fixed
Soil	5–15	10	7–10	8	14.5–17	15
Rock	—	24.5	—	15000	—	45
