# Supplementary material for: Formation of the 2015 Shenzhen landslide as observed by SAR shape-from-shading
Source: Sci Rep. 2017 Mar 3;7:43351. doi: 10.1038/srep43351 (PMC5335704; doi:10.1038/srep43351)
Supplement: Supplementary Information [file srep43351-s1.pdf]

Formation of the 2015 Shenzhen landslide as observed by SAR shape-from-shading

Chisheng Wang[1], Qingquan Li[1*], Jiasong Zhu[1*], Wei Gao[2], Xinjian Shan[3], Jun Song[2], Xiaoli Ding[4]

Data description

We used 27 Cosmo-SkyMed radar images from December 2013 to January 2016 to generate the SAR-SFS DEMs. The COSMO-SkyMed system was founded by the Italian Space Agency and the Italian Ministry of Defence, and consists of four satellites equipped with X-band (3.1 cm wavelength) SAR. The satellite constellation has the capability to obtain radar data with a short revisit time. The data used in this study were from two satellites of the constellation, having a stable observation period of ~1 month. The images were captured in descending track using stripmap HIMAGE operational mode. Each image covers around 40×40 km, having a geometric resolution of ~3 m. To focus on the landfill, we used a ~1.5×1 km sub-area for the SAR-SFS processing.

Supplementary Table 1. Details of the Cosmo-SkyMed radar images

No	Satellite No.	Date	Heading	Polarization	Mode	Side-looking	Incidence angle
1	SAR4	20131207	Descending	HH	STR_HIMAGE	right	32.23°
2	SAR4	20140108	Descending	HH	STR_HIMAGE	right	32.23°
3	SAR4	20140209	Descending	HH	STR_HIMAGE	right	32.23°
4	SAR4	20140313	Descending	HH	STR_HIMAGE	right	32.23°
5	SAR4	20140410	Descending	HH	STR_HIMAGE	right	32.23°
6	SAR2	20140528	Descending	HH	STR_HIMAGE	right	32.23°
7	SAR4	20140617	Descending	HH	STR_HIMAGE	right	32.23°
8	SAR4	20140804	Descending	HH	STR_HIMAGE	right	32.23°
9	SAR4	20140905	Descending	HH	STR_HIMAGE	right	32.23°
10	SAR2	20141003	Descending	HH	STR_HIMAGE	right	32.23°
11	SAR2	20141120	Descending	HH	STR_HIMAGE	right	32.23°
12	SAR4	20141124	Descending	HH	STR_HIMAGE	right	32.23°
13	SAR4	20141226	Descending	HH	STR_HIMAGE	right	32.23°
14	SAR2	20150123	Descending	HH	STR_HIMAGE	right	32.23°
15	SAR4	20150228	Descending	HH	STR_HIMAGE	right	32.23°
16	SAR2	20150328	Descending	HH	STR_HIMAGE	right	32.23°
17	SAR4	20150417	Descending	HH	STR_HIMAGE	right	32.23°
18	SAR4	20150519	Descending	HH	STR_HIMAGE	right	32.23°
19	SAR4	20150531	Descending	HH	STR_HIMAGE	right	32.23°
20	SAR4	20150706	Descending	HH	STR_HIMAGE	right	32.23°
21	SAR4	20150722	Descending	HH	STR_HIMAGE	right	32.23°
22	SAR4	20150807	Descending	HH	STR_HIMAGE	right	32.23°
23	SAR4	20150908	Descending	HH	STR_HIMAGE	right	32.23°
24	SAR4	20151010	Descending	HH	STR_HIMAGE	right	32.23°
25	SAR2	20151123	Descending	HH	STR_HIMAGE	right	32.23°
26	SAR4	20151213	Descending	HH	STR_HIMAGE	right	32.23°
27	SAR4	20160114	Descending	HH	STR_HIMAGE	right	32.23°

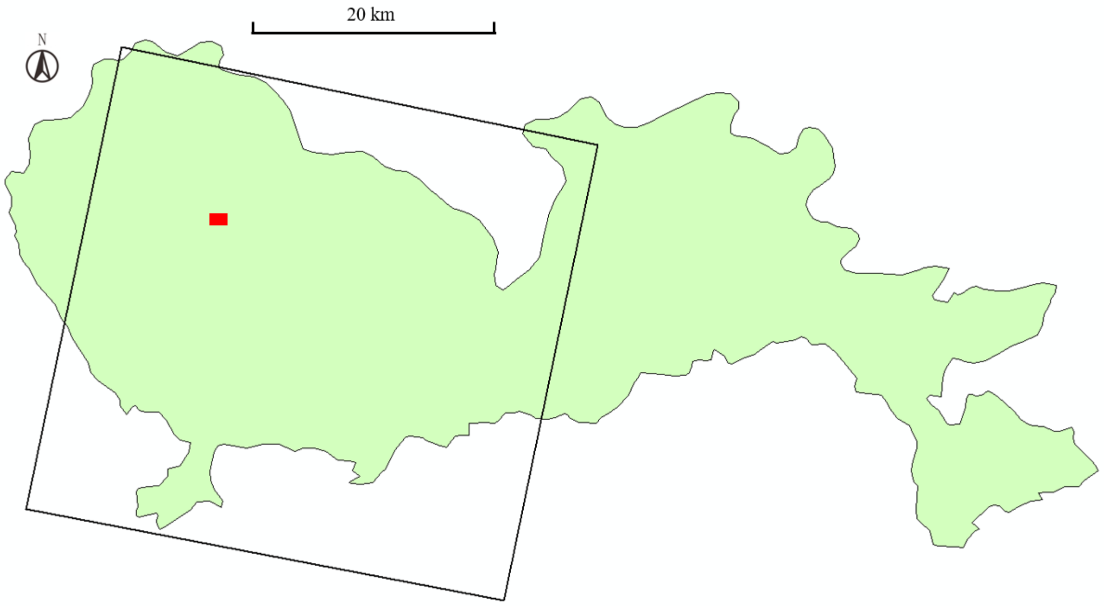

Supplementary Fig. 1. The location of the original SAR image (black rectangle) and the sub-image (red rectangle) used in this study. The figure was generated in Esri ArcMap (ArcMap 10.3 from http://desktop.arcgis.com/zh-cn/arcmap/).

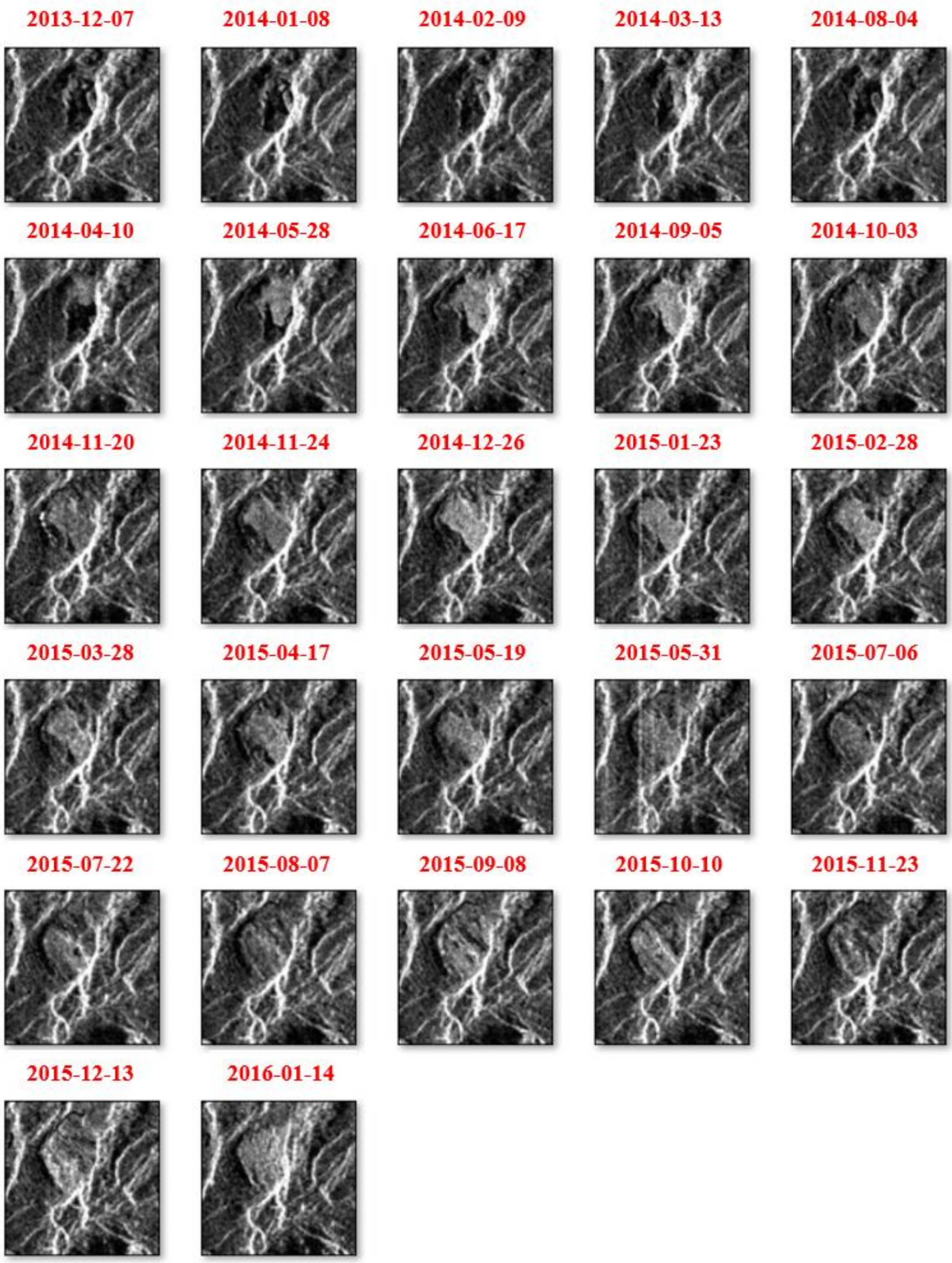

Supplementary Fig. 2. The series of Cosmo-SkyMed radar images from December 07, 2013, to January 14, 2016, showing the evolution of the Hongao landfill site (purchased under license from the Italian Space Agency). In total, 21 images starting from June 17, 2014, were used to generate the DEMs.

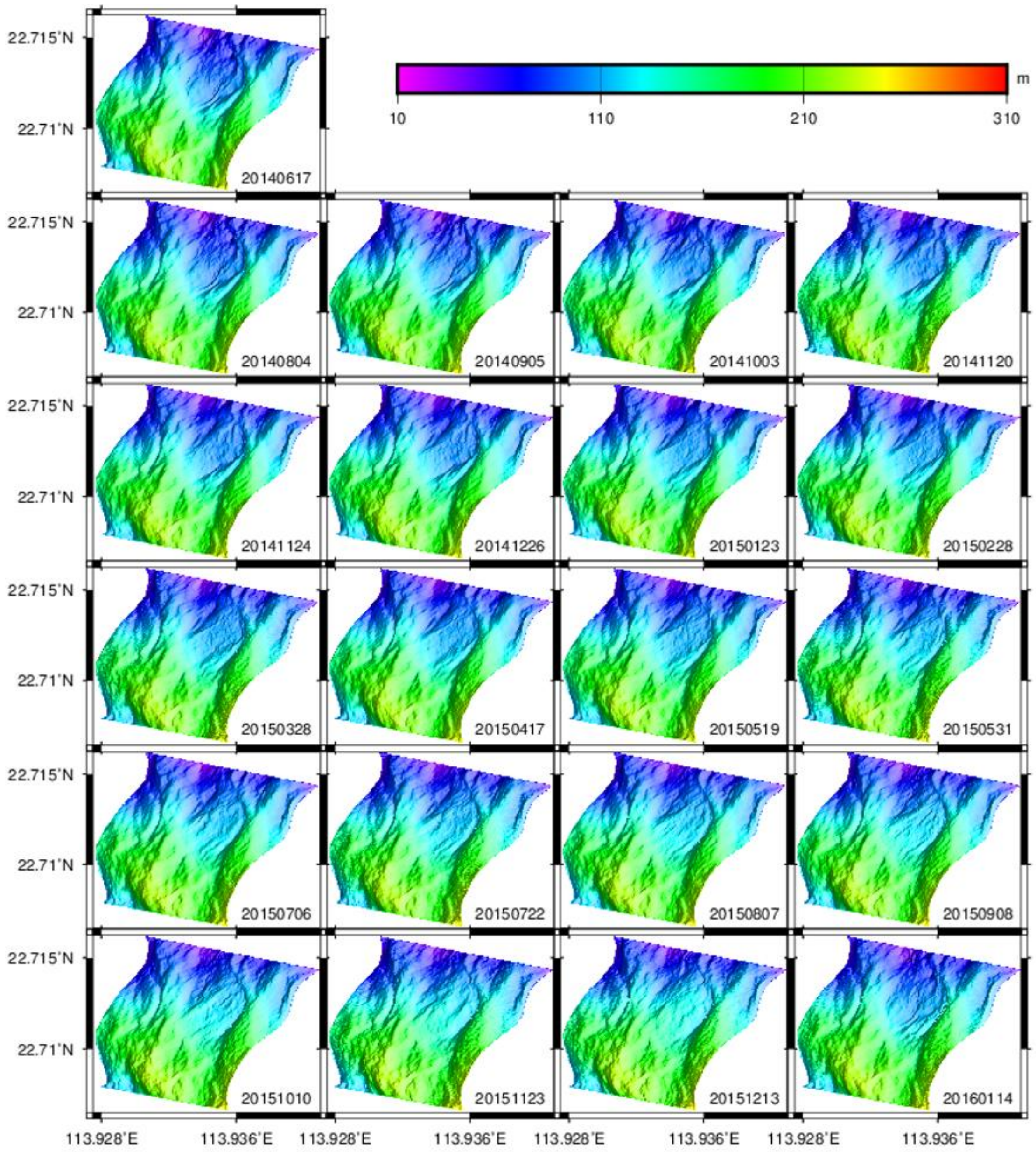

Supplementary Fig. 3. The geo-coded SAR-SFS DEMs from June 17, 2014, to January 14, 2016.

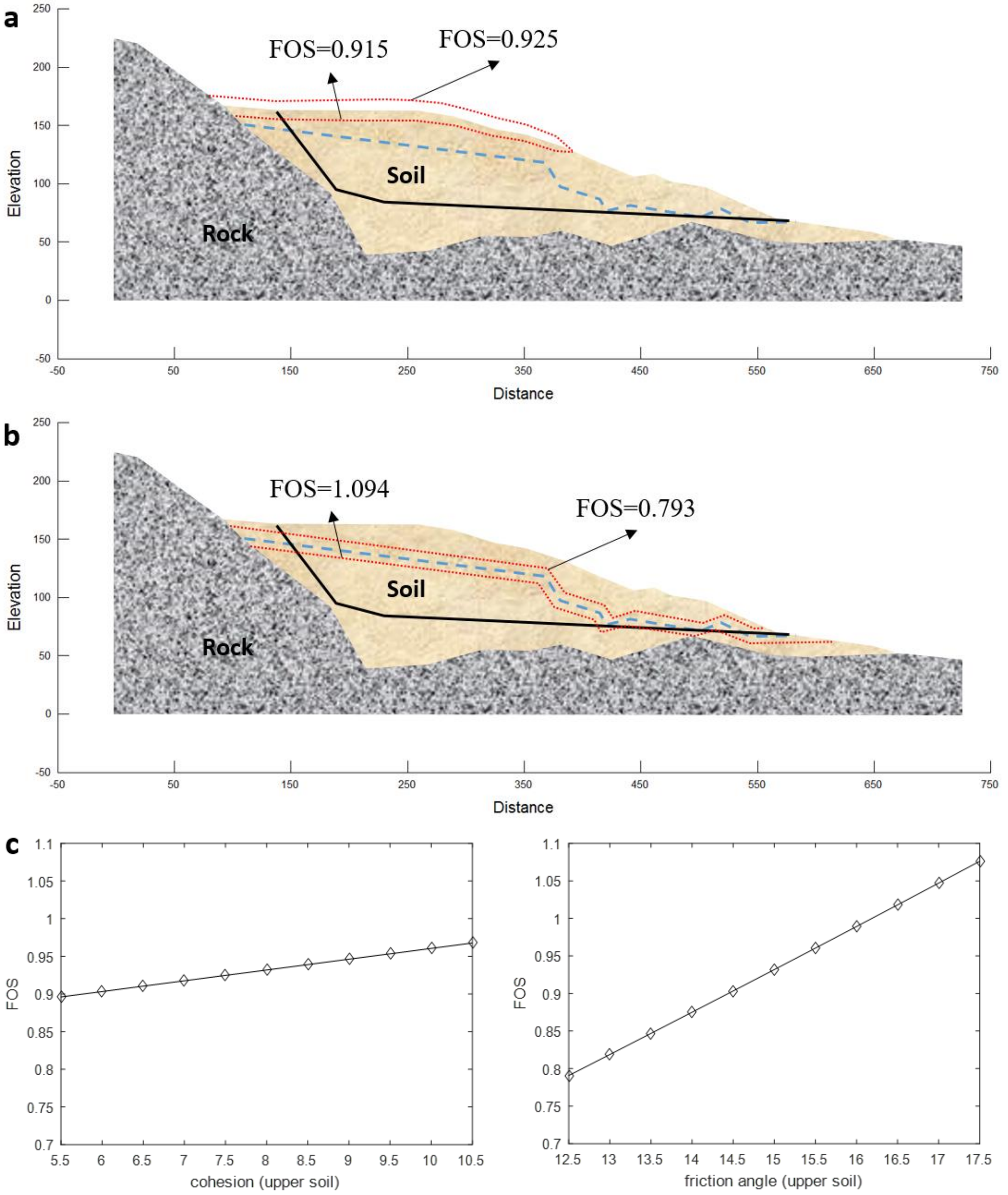

Supplementary Fig. 4. Sensitivity testing of model parameters. (a) Topographic parameter testing. The red dashed lines denote the modified topography profiles, with the corresponding FOS values shown above. (b) Piezometric line parameter testing. The red dashed lines denote the modified piezometric lines, with the corresponding FOS values shown above. (c) Cohesion and friction angle parameter testing.

a

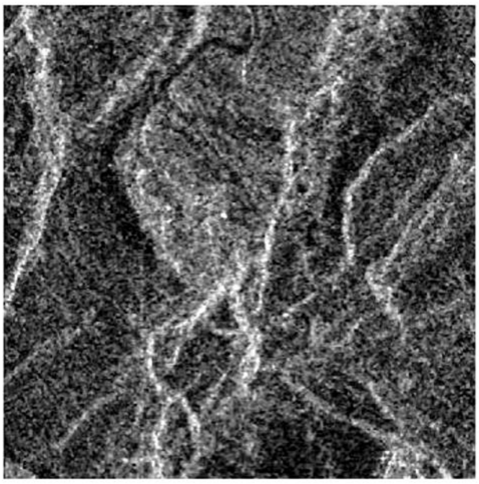

b

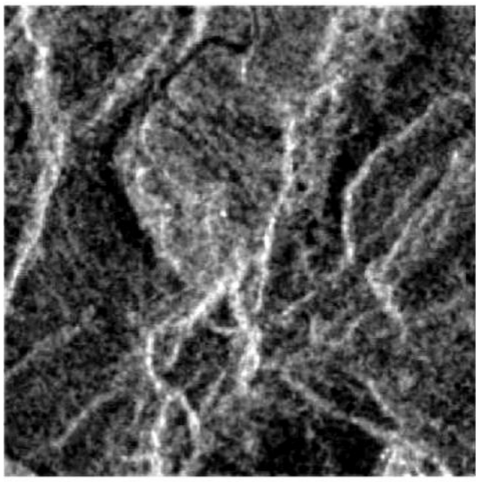

Supplementary Fig. 5. Minimization problem in SAR SFS. (a) SAR observed brightness map. (b) Estimated SAR brightness map using optimized surface gradients.

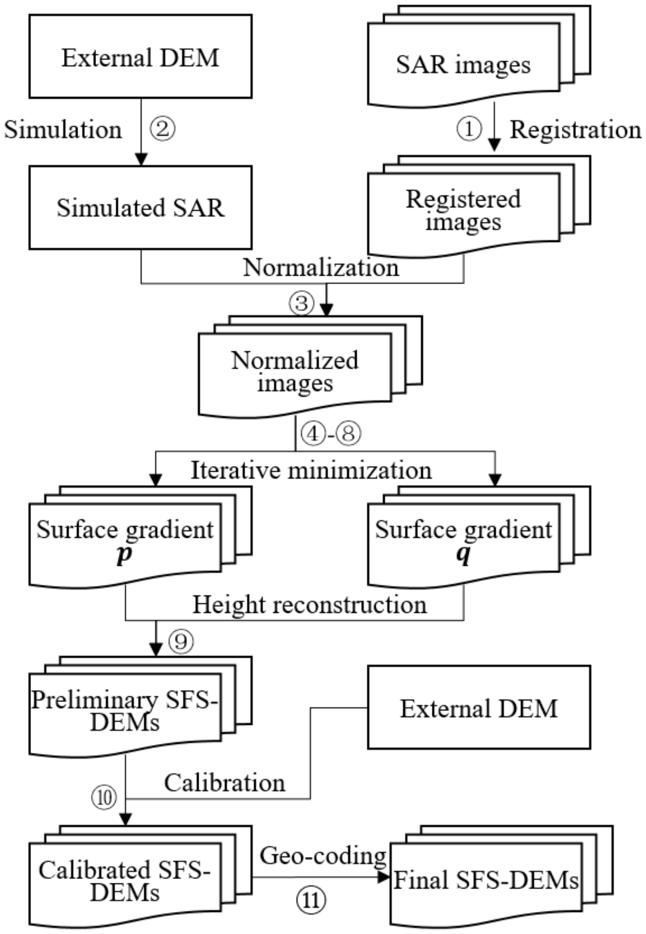

Supplementary Fig. 6. Workflow of the SAR-SFS algorithm. The numbers correspond to the steps mentioned in the algorithm procedure description.
